# How does anonymous online peer communication affect prevention behavior? Evidence from a laboratory experiment

**DOI:** 10.1371/journal.pone.0207679

**Published:** 2018-11-21

**Authors:** Indrani Saran, Günther Fink, Margaret McConnell

**Affiliations:** 1 Boston College School of Social Work, Chestnut Hill, MA, United States of America; 2 Swiss Tropical and Public Health Institute and University of Basel, Basel, Switzerland; 3 Harvard T.H. Chan School of Public Health, Boston, MA, United States of America; Stanford University Medical Center, UNITED STATES

## Abstract

While the importance of social networks for health behaviors is well-recognized, relatively little is known regarding the accuracy of anonymous online communication and its impact on health behavior. In 2012, we conducted a laboratory experiment in Boston, Massachusetts with 679 individuals to understand how anonymous online communication affects individual prevention decisions. Participants had to opt for or against investing in prevention over three sessions, each consisting of 15 experimental rounds. In the third session only, participants could share their experiences with a group of 1–3 other anonymous participants after each round. Groups exchanged an average of 16 messages over the 15 rounds of the third session. 70% of messages contained information about the subject’s prevention decision and the resulting health outcome. Participants were more likely to communicate when they prevented than when they did not, with prevention failures resulting in the highest probability of sending a message. Nonetheless, receiving an additional message reporting prevention increased the odds a subject would prevent by 32 percent. We find that participants tend to adopt the prevention behavior reported by others, with less weight given to the reported outcomes of prevention, suggesting that social networks may influence behaviors through more than just information provision.

## Introduction

A growing body of evidence suggests that social networks may play an important role in people’s health behaviors such as smoking [1], alcohol consumption [2], the types of foods eaten [3], and amount of exercise performed [4]. While some of these correlations within networks could emerge because people tend to associate with those who share similar characteristics or because of common environmental influences [5–7], there is also experimental evidence that social networks may directly influence people’s health behaviors [8–10].

There are two primary channels through which social networks are likely to affect behavior. First, direct information exchange within social networks may enable people to learn about the advantages and disadvantages of engaging in a health behavior. Recent studies suggest that information received from peers related to the efficacy of health technologies can encourage uptake of effective health products such as vaccines [11], insecticide-treated bednets for malaria prevention [12], and artemisinin-based combination therapy for malaria treatment [13].

Second, people may imitate the behavior of their peers because they want to adhere to what they perceive to be the norm within their social networks [14]. Research has shown that providing information to people on how their peers are behaving can influence health behaviors such as alcohol and tobacco use [15,16], dietary patterns [17,18], and physical activity levels [8,19].

In recent years, anonymous online social networks such as health-focused support groups and discussion boards have become an increasingly important source of health information exchange [20,21]. The relatively low costs of participating in online social networks can increase interactions with others [22], and can also allow access to tailored health information [22–24]. However, the anonymous nature of many online social networks may also reduce the reliability of the information being shared [22,23,25], as is often the case with vaccines [26–28]. Informational biases may also result from selective reporting of experiences [29]. Previous reviews have found mixed and generally small effects of online social networks on health behaviors [30–33] and there is little quantitative evidence on the impact of anonymous online information-sharing on health behaviors and health outcomes [34,35].

We used a laboratory experiment to assess the quantity and quality of anonymous health information shared online, as well as the impact of the information shared on the uptake of an illness prevention technology.

## Methods

### Ethics statement

Ethical approval for this study was granted by the Harvard T.H. Chan School of Public Health Internal Review Board (Protocol# 22455–02). Written consent was obtained from all participants.

### Overview of study

The experiment was conducted in 2012 at a university laboratory with 679 adult participants recruited via flyers and emails from the Greater Boston area. The experiment was designed to simulate a setting where individuals need to make decisions about whether to invest a small amount of money in an illness prevention technology which reduces, but does not eliminate, the probability of falling ill (for the purposes of the experiment, we specify this as an income loss to simulate the monetary costs often experienced due to sickness). Each subject participated in 3 sessions consisting of 15 rounds each. Participants were given the following instructions: “You will be playing this game for 15 periods. In each period you will earn an income of US$ 10 if you stay healthy and an income of zero if you fall sick. The probability of falling sick in each period is constant at p = 0.[x] (a [x] in 10 chance) over the game period. In each period you can invest in a preventive health technology for $1 which lowers the probability of falling sick.” [x] was either 3, 5 or 7 depending on the participant (see the section “Randomized Parameters” below). If participants chose to invest in the prevention technology, their incomes when they remained healthy and when they fell sick were $9 and $-1 respectively (see S1 Fig for details on the experimental design).

Participants were recruited by email using the lab's email list (a list of people who had participated in previous studies or expressed interest in doing so) and through Craigslist using the standard procedures of the experimental lab. The experimental session was about one hour and thirty minutes long. All participants received a show-up fee of US$ 10. Participants were informed that they would receive additional payments corresponding to the outcomes of one randomly selected round experienced with each of three sessions. Across the three sessions, participants could thus earn an additional income between $-3 (prevented and got sick all three times) and $+30 (no prevention, never sick). The average payout per person, including the $10 everyone received for participating, was $28.

### Peer messaging

After the first two sessions of independent decision-making, participants were informed in the third session that they would be able to communicate with other players in their group by sending and receiving messages. Each participant was randomly assigned to a group of 1–3 other anonymous participant(s). All group members were present and visible to each other in the computer lab as they participated in the study at the same time. However, they would not have known specifically who among the participants were a part of their messaging group and we generally observed very little communication between subjects before or after the session.

The laboratory was set up for a maximum of 20 participantswho would be randomly grouped into groups of four. The vast majority of groups consisted of 4 people (154/177 groups or 87%), which was the default for the study. Smaller groups only happened if the number of subjects that showed up for a given laboratory session was not divisible by 4. So, for example, if 19 people showed up, there were four groups of 4, and one of group size 3. Overall, there were only 17 (9.6%) groups with 3 people, and 6 (3.4%) groups with 2 people.

At the end of each of the 15 rounds, participants simultaneously sent messages and received messages from other members of their group, using textbox windows.

### Randomized parameters

The experiment randomly varied a number of parameters including the probability the participant fell sick in the absence of prevention (baseline illness rate), the effectiveness of the prevention technology in reducing the probability of falling sick, and the information participants received about the prevention technology. S1 Table includes a summary of the different experimental treatment arms.

The baseline probability of falling sick without prevention was either 0.30, 0.50, or 0.70, was known to all participants, and was the same for all members within a messaging group. The effectiveness of the prevention technology was also randomly assigned with some participants receiving a more effective technology (absolute risk reduction of 20, rather than 12, percentage points). In addition, some participants were randomized to receive public health messaging that encouraged investing in prevention.

### Socio-demographic information

At the end of the session, participants completed a survey which collected demographic information, and details about their usual health and prevention behavior. They were asked if they were currently taking multi-vitamins (yes/no), whether they had gotten a flu shot in the last 12 months (yes/no), whether they believed all children should be vaccinated (yes/no) how many times they had visited a dentist in the past year and how often they use sunscreen (always/often/occasionally/never). The health prevention behavior was collected after the experiment in order to avoid priming the participants’ prevention decisions in the experiment by asking about their usual prevention behavior [36].

### Message coding

We coded all messages exchanged into categories that we expect would have an impact on others’ behavior: messages that provided some information about participants’ experiences with or without prevention and messages that expressed participants’ attitudes towards prevention. We used the following categories for the messages: “Informational”, “Encourages Prevention”, “Discourages Prevention” and “Conversational.” A message was coded as “informational” if it contained at least some information about the subject’s prevention decision and/or illness outcome. When messages contained information, we also coded whether the subject communicated their success or failure with prevention/non-prevention in that particular round (for example if the subject said “I invested and did not get sick”) (S2 Table provides further examples).

Messages were classified as “encouraging” or “discouraging prevention” if they mentioned the advantages of prevention or the disadvantages of prevention, either generally or in reference to this specific prevention technology. “Conversational” messages were those that did not fit into any of the other categories (S2 Table provides further examples). Some messages were coded as both containing information and either encouraging prevention or discouraging prevention. Although the coding of the messages was performed by a single author, two co-authors independently re-coded 10% (280 messages) each in order to check the reliability of the coding. Across all types of messages, there was 94% agreement in categorization between the two coders (for both sets of messages that were re-coded). In cases of disagreement, the final coding decision was jointly made by the author team.

Using the information contained in the messages on whether the subject prevented in that round and the illness outcome, we calculated, by group, the reported illness rates with and without prevention. We compared these reported rates to the expected illness rates, given the assigned probability of falling ill in the absence of prevention, and the risk reduction when using the prevention technology. Since the baseline probabilities of falling ill varied by subject, we re-scaled all reported rates so that the expected probability of falling ill in the absence of prevention was 0.5, and the expected probability of falling ill with prevention was 0.34 (the mean expected absolute risk reduction within all group was 16 percentage points)

### Analytical approach

In a first step, we used multivariable logistic models to assess the determinants of posting a message online in the third session. The primary variable of interest in this first step were participants’ experiences (utilization of prevention and outcome) as well as the messaging behavior of others. In the regression models we controlled for the age, gender, marital status, parental status, education, income, ethnicity, occupation, usual prevention behavior of the subject, the public health messaging the subject received, the effectiveness of their randomly assigned preventive technology, and their randomly assigned baseline illness rate. Usual prevention behavior included whether the subject took vitamins, had a flu shot in the past year, favored child vaccination, the number of dentist visits she/he had in the past year, and whether the subject regularly used sunscreen. Standard errors were adjusted for clustering at the group level.

In a second step, we used multivariable logistic models with session and participant fixed effects to assess the effect of online messages on prevention. We included all three sessions in this analysis and each observation consisted of a subject-round. Our outcome of interest was a binary variable for whether a participant prevented in a given round. Our main independent variable in this analysis was the number of messages the participant received in the previous round which was coded as zero for all rounds in the first two sessions. We included participant fixed effects in order to account for individual-level factors that could influence the propensity to send messages as well as their propensity to engage in prevention. These regression models also controlled for the effectiveness of the prevention technology which varied for participants across the three sessions and included session fixed effects to control for general learning. Standard errors were adjusted for clustering at the individual level.

All analyses were conducted using Stata/SE version 14.0 (StataCorp, College Station, TX) [37].

## Results

### Characteristics of study participants

Table 1 provides detailed descriptive statistics on the study sample. Participants were, on average, 31 years old. 52% of participants were male, 54% were white, 14% black, and 6% Hispanic. Only 11% were married and 14% had children. 53% of individuals were currently enrolled as university students. Accordingly, participants were on average highly educated (52% had a college degree or higher) with relatively low income—only 17% of participants had an annual income above $40,000. Given the high proportion of college students in our sample, however, the distribution of income may not accurately represent the socio-economic background of our participants.

**Table 1:** Descriptive statistics of lab experiment participants (N = 679).

	No.	% or Mean ±SD
**Demographics**		
Age		31.2 ± 13.6
Male	350	51.5%
Married	76	11.2%
Has Children	93	13.7%
**Education**		
Some High School or Less	8	1.2%
Completed High School (or equivalent)	41	6.0%
Some College	276	40.6%
College Diploma	151	22.2%
Some graduate school	72	10.6%
Graduate Degree	127	18.7%
Refused to Answer	4	0.6%
**Ethnicity**		
African American	95	14.0%
Hispanic	40	5.9%
White/Caucasian	365	53.8%
Asian American	92	13.5%
Other	63	9.3%
Refused to Answer	24	3.5%
**Occupation**		
Student	358	52.7%
Other	237	34.9%
Retired	14	2.1%
Unemployed	58	8.5%
Refused to Answer	12	1.8%
**Income**		
$0	91	13.4%
$1–19,999	319	47.0%
$20,000–39,999	119	17.5%
$40,000–59,999	61	9.0%
$60,000–79,999	30	4.4%
$80,000 or more	25	3.7%
Refused to Answer	34	5.0%
**Prevention Behavior**		
Takes Vitamins	294	43.3%
Had Flushot in Last Year	318	46.8%
Believes All Children should be Vaccinated	599	88.2%
Times Saw Dentist in Last Year		1.3 ± 1.2
Uses Suncreen Often/Always	264	38.9%

In terms of their general prevention behavior, individuals reported a mean of 1.3 dentist visits over the past year and 88% believed that all children should vaccinated. However, only 39% used sunscreen “often” or “always”, 44% were taking multi-vitamins, and 47% had had a flu shot in the past year. A slightly higher proportion of our sample reported that they engage in these prevention behaviors than the population as a whole [38–41].

### Baseline prevention behavior

In the first two sessions, individuals independently made their decision about whether to invest in prevention. In these two sessions the mean prevention rate was 70%. Only 2% of participants (14/679) never prevented in these two sessions, while 25% of participants (170/679) prevented in every round.

### Messaging behavior

In the last session, participants were able to share information with peers in their group. The average group size was 3.8 and the vast majority of groups (87%) consisted of four participants. Approximately 60% of participants (82% of groups) sent at least one message over the 15 rounds of the third session, and individuals (groups) sent, on average, 4.2 (16) messages each. This resulted in individuals’ receiving, on average, 12 messages over the 15 rounds. The distribution of the number of messages sent by groups is shown in S2 Fig.

Table 2 shows the multivariable regression results for messaging behavior in the third session. The coefficients on both a baseline illness rate of 0.5 and 0.7 (relative to the reference group with a baseline rate of 0.3) are large and statistically significant, suggesting that the general messaging propensity strongly increases with the risk of falling sick. Columns 2 and 3 of Table 2 show the associations between participants’ prevention experiences and messaging behavior. The reference group in both cases is participants who did not prevent and did not fall sick in the previous round. In the first round (Column 2), when participants had not yet received any messages from others, only those who prevented and fell sick had a marginally significant higher odds of sending a message compared to the reference group. In rounds 2–15 (Column 3), previous prevention without getting sick was associated with increased odds of sending a message (OR = 1.47, 95% CI [1.12 1.94]), and those who prevented and fell sick were even more likely to send a message. In terms of the direct response to other messages, each additional message received from others in the previous round was associated with 3.46 times the odds (95% CI [3.05 3.92]) of sending a message.

**Table 2:** Predictors of message-sending using logistic regressions.

	Outcome: Odds of Sending a Message		
	(1)	(2)	(3)
A. Received Public Health Message	1.12	0.78	1.2
	[0.85,1.49]	[0.52,1.16]	[0.91,1.60]
B. Received More Effective Prevention Technology	1.06	0.92	1.03
	[0.81,1.38]	[0.63,1.35]	[0.79,1.35]
C. Baseline Illness Rate = 0.30	Ref. Group	Ref. Group	Ref. Group
D. Baseline Illness Rate = 0.50	2.91**	2.11**	1.75**
	[2.08,4.09]	[1.28,3.48]	[1.26,2.44]
E. Baseline Illness Rate = 0.70	4.61**	3.82**	2.01**
	[3.27,6.48]	[2.33,6.27]	[1.42,2.85]
F. Prevented		0.83	1.47**
		[0.43,1.62]	[1.12,1.94]
G. Fell Sick		0.62	0.88
		[0.27,1.44]	[0.67,1.16]
H. Prevented X Fell Sick		2.3	1.46*
		[0.89,5.96]	[1.06,2.00]
I. Number of Messages Received in Previous Round			3.46**
			[3.05,3.92]
Rounds	All	1st Only	Rounds 2–15
Mean of Dependent Variable in Reference Group (No Prevention, Not Sick)	0.28	0.20	0.18
		
		
P-value: G+H = 0		0.14	0.00
Number of Observations	10185	679	9506

Notes: Table shows logistic regression results for predictors of messaging overall (Column 1) after the first round only (Column 2), and after rounds 2–15 (Column 3). All regressions include the following controls: the age, gender, marital status, ethnicity, occupation, parental status, education, income, and prevention behavior of the individual (takes vitamins had a flu shot in the past year, favors child vaccination, number of dentist visits in past year, sunscreen use). Coefficients are expressed in terms of odds ratios and 95% confidence intervals are in brackets. Standard errors are adjusted for clustering by individual.

*p<0.05

**p<0.01

**Fig 1:**
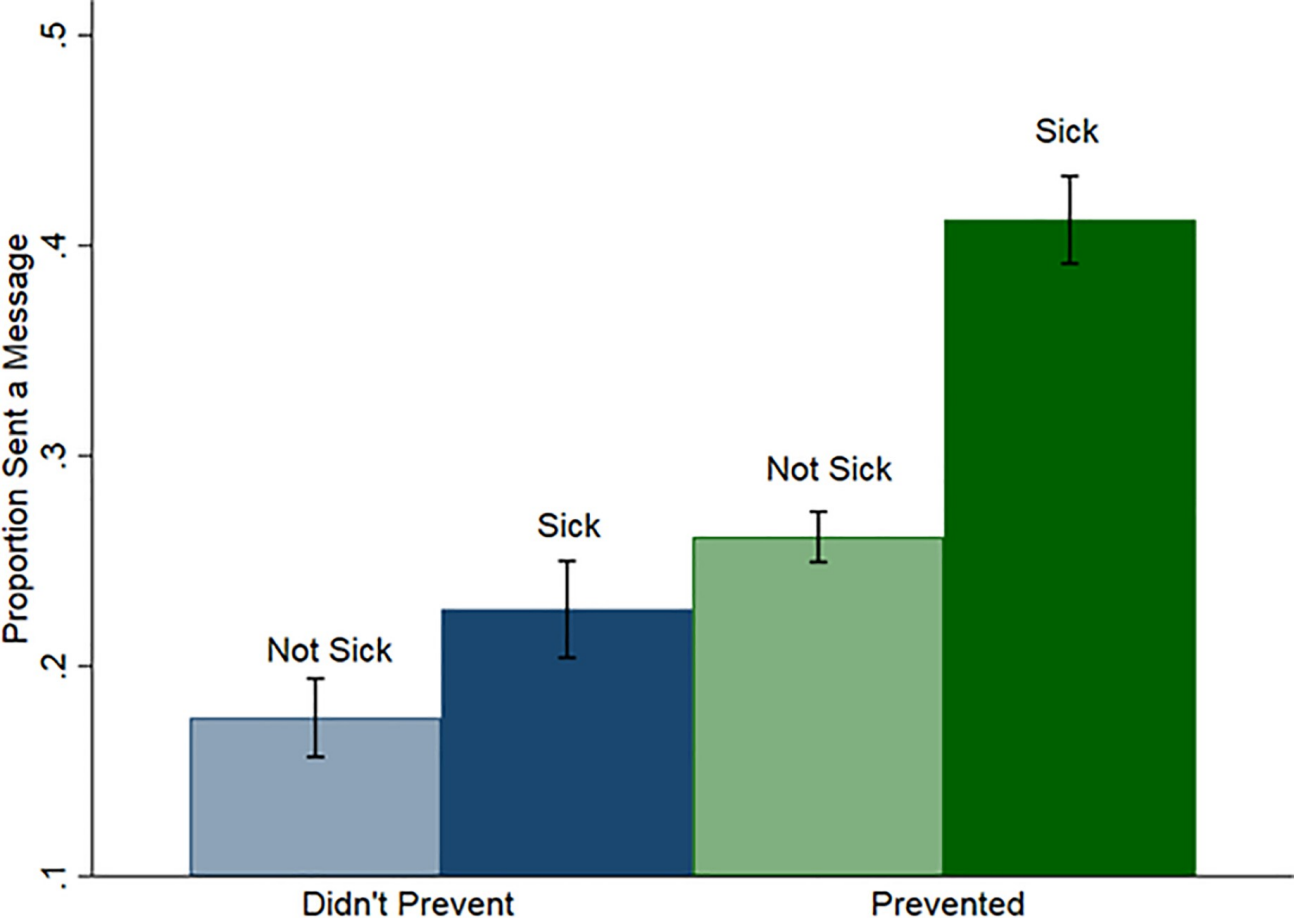
Message-sending by prevention decision and outcome. Probability of sending a message by whether the individual prevented and whether they fell sick. Error bars indicate 95% confidence intervals.

Fig 1 displays the proportion of people who sent a message, across all 15 rounds of the third session, by whether they prevented, and whether they fell sick. Overall, people who prevented were more likely to send a message, as were people who fell sick. The highest messaging rates were among people who prevented but had adverse health outcomes.

### Message content

**Fig 2:**
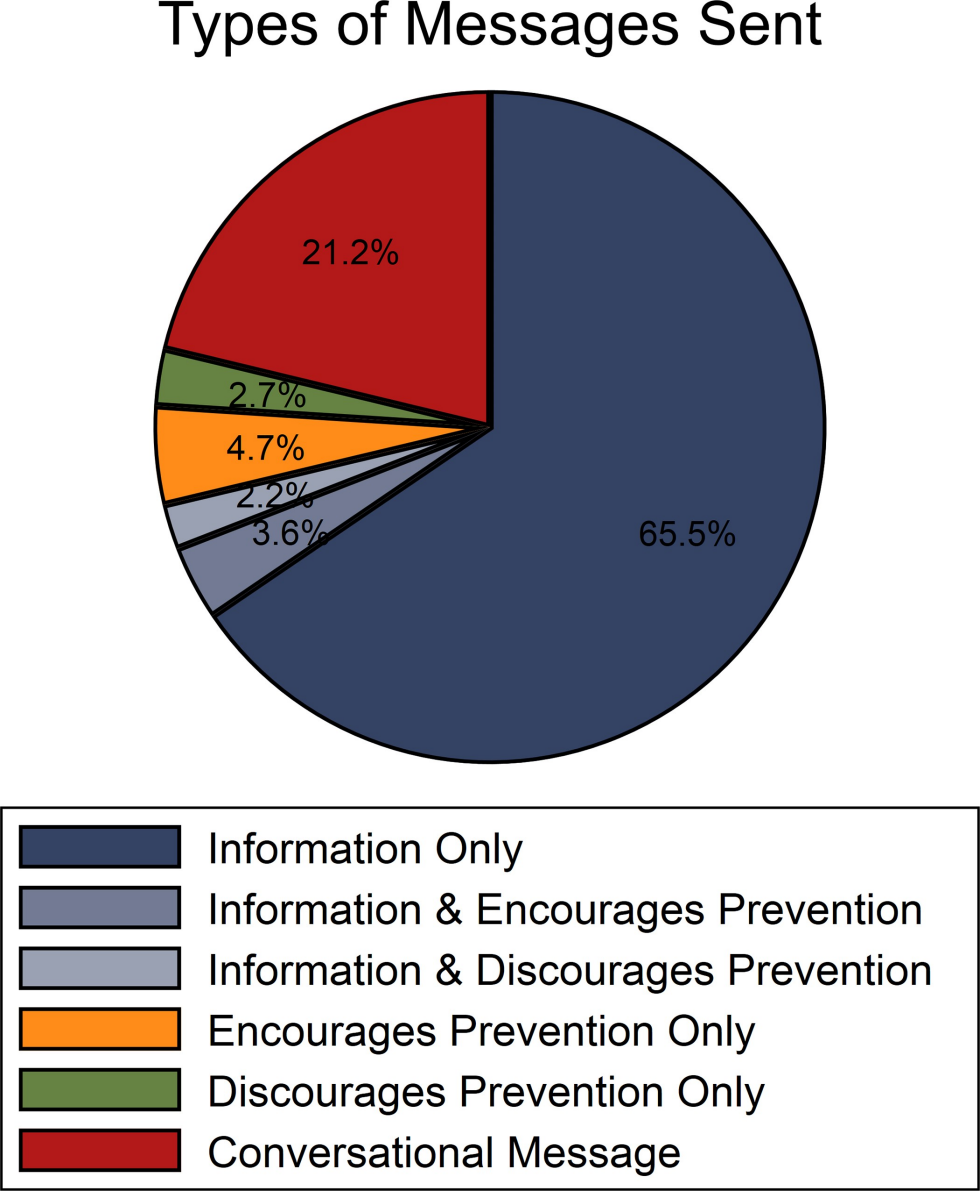
Types of messages sent by individuals over 15 rounds. Messages either provided information about the prevention decision and result, encouraged or discouraged prevention, or were unrelated to the prevention decision (conversational message).

Fig 2 summarizes the content of the 2806 messages that were sent over the 15 rounds. Approximately 70% of messages contained some information about the current and/or previous rounds, 8% of messages directly encouraged others to invest in prevention, and nearly 5% discouraged prevention. 21% of messages were conversational and unrelated to the laboratory experiment or to prevention.

**Fig 3:**
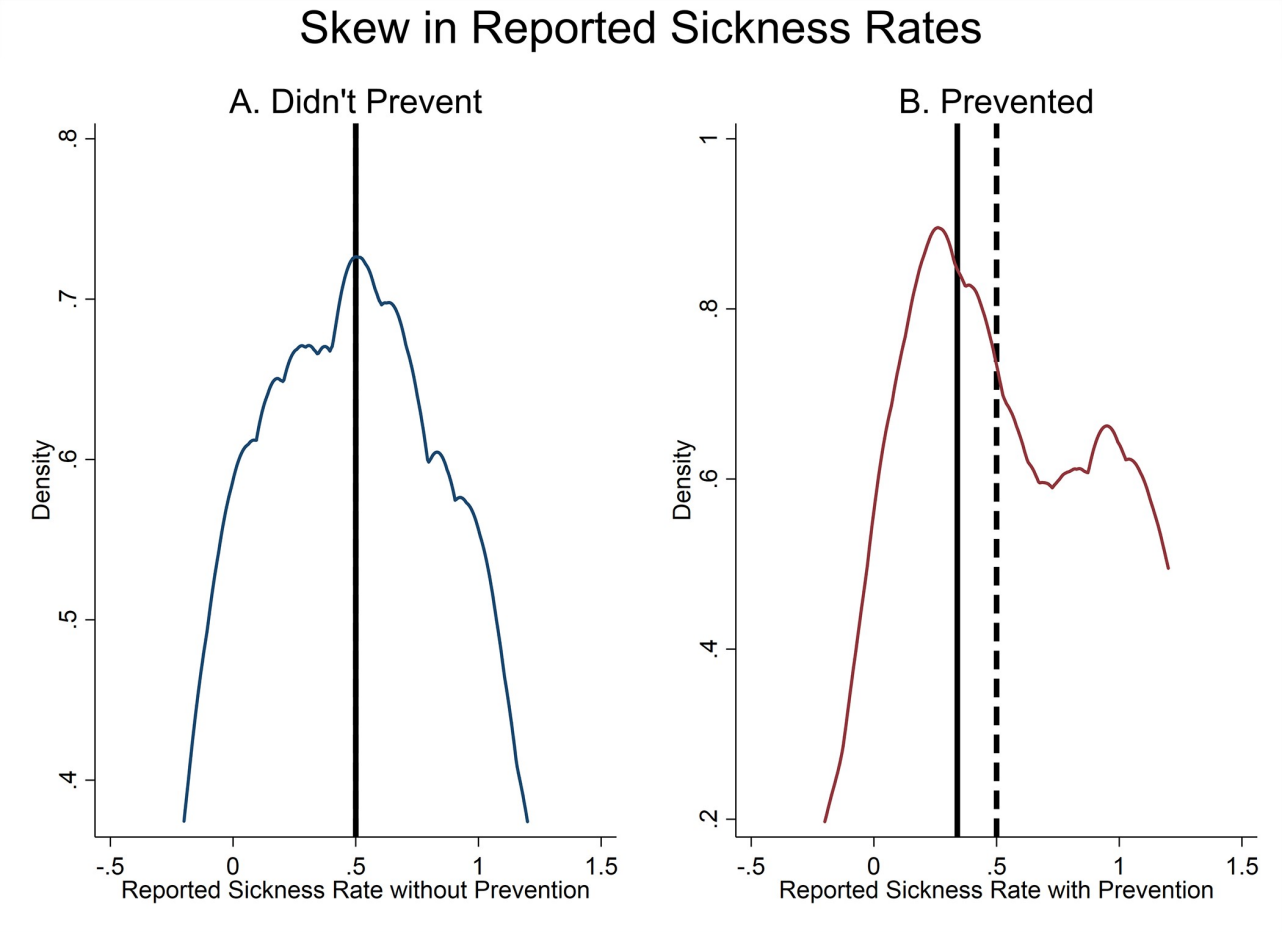
Reported illness rate with and without prevention. Figure shows the distribution, by group, of reported illness rates when not preventing (Panel A) and when preventing (Panel B). Reported illness rates were re-scaled so that the expected probability of falling sick is 0.5 in the absence of prevention and 0.34 with prevention. In Panel A, sample is limited to the 55/177 (31%) groups who reported on non-prevention outcomes at least once, and in Panel B the sample is limited to the 97/177 groups (55%) who reported on prevention outcomes at least once. Solid line indicates expected illness rate while dashed line indicates the median reported illness rate (lines are overlapping in Panel A).

In Fig 3, we use the information contained in the messages to plot the distribution, across groups, of participants’ reported illness rates when they did and did not use the prevention technology. In the 55/177 groups (31%) where a subject reported on non-prevention outcomes at least once, the median reported re-scaled illness rate without prevention (0.5) is approximately the same as the expected illness probability without prevention (Fig 3, Panel A). However, in the 97/177 groups (55%) where participants reported on prevention outcomes at least once, the median reported re-scaled illness rate with prevention was 0.16 points higher than the expected illness probability, which means that prevention on average looks substantially less effective than it is (0.5 median illness risk with reported prevention compared to the expected probability of 0.34) (Fig 3B).

### Messaging and prevention

Table 3 presents the logistic regression results for the impact of messaging on prevention uptake. On average, across all three sessions, participants prevented 71% of the time. Conditional on individual and session fixed effects, each additional message received in the previous round was associated with a 9% increase in the odds of preventing in the current round (95% CI [0.98 1.21]) (Column 1). Receiving a message that reported on the sender’s prevention decision and outcome increased the odds of prevention by 14% (95% CI [1.01 1.29]). (Column 2)

**Table 3:** Effect of messages received in previous round on prevention in current round.

	Outcome: Probability of Prevention in Current Round			
	(1)	(2)	(3)	(4)
A. Number of Messages Received in Previous Round	1.09			
	[0.98,1.21]			
B. Number of Messages in Previous Round Reporting Prevention Decision and Outcome		1.14*		
		[1.01,1.29]		
C.Number of Successful Prevention Messages Received in Previous Round			1.53**	
			[1.25,1.86]	
D. Number of Failed Prevention Messages Received in Previous Round			1.1	
			[0.91,1.33]	
E. Number of Successful Risk Messages Received in Previous Round			0.57**	
			[0.42,0.79]	
F. Number of Failed Risk Messages Received In Previous Round			0.94	
			[0.69,1.27]	
G. Number of Messages Received in Previous Round Reporting Prevention				1.32**
				[1.15,1.52]
H. Number of Messages Received in Previous Round Reporting Risk-Taking				0.76*
				[0.60,0.96]
I. Number of All Other Messages Received in Previous Round		1	1.01	1
		[0.86,1.16]	[0.89,1.16]	[0.88,1.15]
P value: C = D			0.01	
P value: E = F			0.01	
P value: G = H				0.00
Mean of Dependent Variable	0.71	0.71	0.71	0.71
Number of Observations	30555	30555	30555	30555

Notes: Results are from logit regressions estimating the association between messages and information received in the previous round and the odds of prevention in the current round, with a fixed effect for each individual (each observation consists of an individual-round). All regressions control for the effectiveness of the randomly assigned preventive technology. The sample includes observations from all sessions although individuals only had the opportunity to receive messages in the third session. The “All other Messages Received” category varies with each regression. Coefficients are in terms of odds ratios and 95% confidence intervals, based on standard errors clustered at the individual level, are in brackets.

*p<0.05

**p<0.01

In Column 3, we divide these informational messages further into those that reported successful (healthy) or unsuccessful (sick) outcomes with prevention and non-prevention (“risk-taking”). Receiving a report of successful prevention in the previous round was associated with a 53% increase in the odds of prevention in the current round (95% CI [1.25 1.86]). However, reports of failed prevention were also associated with a slightly increased, though statistically non-significant, odds of prevention (OR = 1.10, 95% CI [0.91 1.33]). Reports of successful risk-taking were associated with a 43% reduction in the odds of prevention (95% CI [0.42 0.79]) but there was no statistically significant association between reports of failed risk-taking and prevention (OR = 0.94, 95% CI [0.69 1.27]). Column 4 considers all reports of prevention and reports of risk-taking, regardless of the illness outcome, or even whether the outcome even reported. An additional message received reporting prevention was associated with 32% higher odds of prevention (95% CI [1.15 1.52]) and each additional message per round reporting risk-taking was associated with 24% lower odds of prevention (95% CI [0.60 0.96]) (Table 3, Column 4).

## Discussion

This study used a laboratory experiment to investigate the impact of anonymous online communication on prevention behavior. The experiment has three main findings. First, consistent with recent evidence [42,43], we find that a large fraction of participants took the opportunity to communicate anonymously in the online platform.

Second, we find that participants were most likely to send a message when they experienced an adverse outcome with the prevention technology, thus resulting in a slightly skewed reporting of health outcomes with prevention. Recent evidence suggests that the quality of health information available online is mixed [23,44,45]. Our evidence indicates that even if individuals share accurate data, selective reporting could result in the aggregate information being biased.

Our third main finding is that despite the reporting bias in favor of failed prevention, online information-sharing is associated with higher prevention rates. These positive impacts appear to be both the result of higher messaging frequency among participants who prevented (as shown in Fig 1 and Table 2), and a tendency for individuals to imitate the behavior of others rather than respond only to reported effectiveness. That is, we find that participants were more likely to prevent when they received reports of others preventing, regardless of the outcome, and participants were less likely to prevent when others reported not preventing, regardless of the outcome. Participants were most likely to engage in prevention when other participants reported to have successfully prevented in the previous period, and least likely to prevent when others reported having successfully not prevented, which shows that information on outcomes also plays an important role in adoption of a new technology. However, even failed prevention reports result in marginally higher propensities to engage in prevention which suggests that imitation is an important driver of behavior in this setting.

Overall, the results presented are consistent with recent evidence that people both imitate others’ behavior and respond to the expected benefits of the action, though we find that the first channel dominates the second in our setting [11–14,14]. Our results are also consistent with a recent online experiment examining adoption of a new application [46]. The authors found that invitations from friends to use the application—which could be considered an endorsement of the product—had a larger effect on adoption than simple notifications that their peers were using the application [46].

Our finding that the actions of peers have more influence on health behaviors than information on effectiveness may offer insight into why public health messaging often has small impacts on people’s behaviors [47,48]. Our experiment is most relevant for understanding individuals‘ prevention behaviors when the health outcome can be observed shortly after investment in the prevention technology and adverse outcomes are relatively frequent regardless of prevention. These include, for example, flu shots, flossing to prevent cavities or handwashing to reduce the risk of foodborne illnesses. In other cases, such as the use of sunscreen, the true benefits of using prevention may not be visible for decades. The imperfect connection between the prevention behavior and the outcome could help explain why people are more responsive to others’ prevention behaviors rather than the impact it had on their health. Our results may be less applicable for preventive behaviors such as wearing sunscreen, where the time frames are much longer and adverse health outcome being targeted is uncommon.

There are some limitations to this study. First many of the participants in this study were students and relatively well-educated and their prevention behavior and response to messaging may not necessarily be generalizable to other populations. Relatedly, the groups in this study were relatively small whereas most online communication platforms reach many thousands of people. In addition, this experiment was conducted with a hypothetical prevention technology. People may behave differently when confronted with the choice of investing in an actual prevention technology. People may also behave differently when their behavior is not being observed by researchers. Lastly, the effect of communication on health behaviors may be different when individuals are able to select the peer group with whom they communicate, particularly if they share common traits [49].

However, laboratory experiments also offer several advantages in studying online behavior: first the decision-making environment is tightly controlled, allowing us to determine how variation in different parameters affect messaging behavior. Second, peer groups in the experiment were randomly assigned which limits the likelihood that peer effects are due to common unobserved factors among the groups [50]. Third, by recording the information shared among participants we can determine both the accuracy of the information shared as well as how it influences behavior. Laboratory experiments have been previously used to study a number of different aspects of social learning [51–53].

## Conclusion

As people increasingly turn to online sources for health information, there is growing interest in finding ways to harness social networks for improving health behaviors while limiting the negative impacts of mis-information. Our results suggest that online communication platforms do not have to present unbiased information in order to have positive effects on health behaviors. In some contexts however, the tendency towards imitating others’ health behaviors could have negative health impacts; for example social norms may drive the overuse of antibiotics [54]. This problem may be exacerbated if people select social networks that offer them a skewed sample of experiences [55,56]. One potential policy implication of our results is that encouraging people to report on social networks when they have invested in prevention behaviors could encourage others to do the same, though the effectiveness of this strategy would need to be studied further. More work is also needed to understand where people seek information on health and how the source of information influences their behavior.

## Supporting information

S1 Dataset Data on prevention and messaging from laboratory experiment. Individual data on prevention from all three sessions of laboratory experiment and on messaging from third session.
(DTA)

S1 Table Summary of experimental treatment arms. Table indicates sample size within each of the experimental treatment groups. There was also a cross-cutting randomization where some subjects received a public health message about prevention. All subjects received the following prompt: *“[]*..*you will be playing this game for 15 periods*. *In each period you will earn an income of US$ 10 if you stay healthy*, *and a income of zero if you fall sick*. *The probability of falling sick in each period is constant at p = [x] (a [x] in 10 chance) over the game period*. *In each period you can invest in a preventive health technology for $1 which lowers the probability of falling sick*.”
(DOCX)

S2 Table Examples of types of messages sent. All messages were coded into one of these four categories. Some messages were counted as both “informational” and either “encourages prevention” or “discourages prevention.”
(DOCX)

S1 Fig Summary of experimental design. The probability p that a person fell ill when they did not prevent (p) was either 0.3, 0.5 or 0.7. The probability that a person fell when they prevented (p’) was lower than the probability of falling ill when they didn’t prevent (p’<p).
(TIF)

S2 Fig Distribution of messages sent. Distribution of total number of messages sent by all individuals in a group (N = 4) over 15 rounds.
(TIF)

## References

[1] Christakis NA, Fowler JH. The Collective Dynamics of Smoking in a Large Social Network. New England Journal of Medicine. 2008;358: 2249–2258. 10.1056/NEJMsa0706154 18499567 10.1056/NEJMsa0706154 PMC2822344

[2] Rosenquist JN, Murabito J, Fowler JH, Christakis NA. The spread of alcohol consumption behavior in a large social network. Ann Intern Med. 2010;152: 426–433, W141. 10.7326/0003-4819-152-7-201004060-00007 20368648 10.1059/0003-4819-152-7-201004060-00007 PMC3343772

[3] Pachucki MA, Jacques PF, Christakis NA. Social Network Concordance in Food Choice Among Spouses, Friends, and Siblings. American Journal of Public Health. 2011;101: 2170–2177. 10.2105/AJPH.2011.300282 21940920 10.2105/AJPH.2011.300282 PMC3222397

[4] Aral S, Nicolaides C. Exercise contagion in a global social network. Nature Communications. 2017;8: 14753 10.1038/ncomms14753 28418379 10.1038/ncomms14753 PMC5399289

[5] Lyons R. The Spread of Evidence-Poor Medicine via Flawed Social-Network Analysis. Statistics, Politics, and Policy. 2011;2 10.2202/2151-7509.1024

[6] Shalizi CR, Thomas AC. Homophily and Contagion Are Generically Confounded in Observational Social Network Studies. Sociological Methods & Research. 2011;40: 211–239. 10.1177/0049124111404820 22523436 10.1177/0049124111404820 PMC3328971

[7] Noel H, Nyhan B. The “unfriending” problem: The consequences of homophily in friendship retention for causal estimates of social influence. Social Networks. 2011;33: 211–218. 10.1016/j.socnet.2011.05.003

[8] Zhang J, Brackbill D, Yang S, Centola D. Efficacy and causal mechanism of an online social media intervention to increase physical activity: Results of a randomized controlled trial. Preventive Medicine Reports. 2015;2: 651–657. 10.1016/j.pmedr.2015.08.005 26844132 10.1016/j.pmedr.2015.08.005 PMC4721409

[9] Centola D. The Spread of Behavior in an Online Social Network Experiment. Science. 2010;329: 1194–1197. 10.1126/science.1185231 20813952 10.1126/science.1185231

[10] and the Look AHEAD Home Environment Research Group, Gorin AA, Wing RR, Fava JL, Jakicic JM, Jeffery R, et al Weight loss treatment influences untreated spouses and the home environment: evidence of a ripple effect. International Journal of Obesity. 2008;32: 1678–1684. 10.1038/ijo.2008.150 18762804 10.1038/ijo.2008.150 PMC2730773

[11] Rao N, Mobius MM, Rosenblat T. Social networks and vaccination decisions. 2007; Available: http://papers.ssrn.com/sol3/papers.cfm?abstract_id=1073143

[12] Dupas P. Short-Run Subsidies and Long-Run Adoption of New Health Products: Evidence From a Field Experiment. Econometrica. 2014;82: 197–228. 10.3982/ECTA9508 25308977 10.3982/ECTA9508 PMC4193678

[13] Learning Adhvaryu A., Misallocation, and Technology Adoption: Evidence from New Malaria Therapy in Tanzania. The Review of Economic Studies. 2014;81: 1331–1365. 10.1093/restud/rdu020 25729112 10.1093/restud/rdu020 PMC4341843

[14] Nolan JM, Schultz PW, Cialdini RB, Goldstein NJ, Griskevicius V. Normative Social Influence is Underdetected. Personality and Social Psychology Bulletin. 2008;34: 913–923. 10.1177/0146167208316691 18550863 10.1177/0146167208316691

[15] Lewis MA, Neighbors C. Social Norms Approaches Using Descriptive Drinking Norms Education: A Review of the Research on Personalized Normative Feedback. Journal of American College Health. 2006;54: 213–218. 10.3200/JACH.54.4.213-218 16450845 10.3200/JACH.54.4.213-218 PMC2459316

[16] Mead EL, Rimal RN, Ferrence R, Cohen JE. Understanding the sources of normative influence on behavior: The example of tobacco. Social Science & Medicine. 2014;115: 139–143. 10.1016/j.socscimed.2014.05.030 24910005 10.1016/j.socscimed.2014.05.030 PMC4124724

[17] Pelletier JE, Graham DJ, Laska MN. Social Norms and Dietary Behaviors among Young Adults. American Journal of Health Behavior. 2014;38: 144–152. 10.5993/AJHB.38.1.15 24034689 10.5993/AJHB.38.1.15 PMC3876876

[18] Robinson E, Thomas J, Aveyard P, Higgs S. What Everyone Else Is Eating: A Systematic Review and Meta-Analysis of the Effect of Informational Eating Norms on Eating Behavior. Journal of the Academy of Nutrition and Dietetics. 2014;114: 414–429. 10.1016/j.jand.2013.11.009 24388484 10.1016/j.jand.2013.11.009

[19] Priebe CS, Spink KS. Using Messages Promoting Descriptive Norms to Increase Physical Activity. Health Communication. 2012;27: 284–291. 10.1080/10410236.2011.585448 21899404 10.1080/10410236.2011.585448

[20] Ancker JS, Carpenter KM, Greene P, Hoffman R, Kukafka R, Marlow LAV, et al Peer-to-Peer Communication, Cancer Prevention, and the Internet. Journal of Health Communication. 2009;14: 38–46. 10.1080/10810730902806760 19449267 10.1080/10810730902806760 PMC3645318

[21] Griffiths F, Cave J, Boardman F, Ren J, Pawlikowska T, Ball R, et al Social networks–The future for health care delivery. Social Science & Medicine. 2012;75: 2233–2241. 10.1016/j.socscimed.2012.08.023 22985490 10.1016/j.socscimed.2012.08.023

[22] Moorhead SA, Hazlett DE, Harrison L, Carroll JK, Irwin A, Hoving C. A new dimension of health care: systematic review of the uses, benefits, and limitations of social media for health communication. J Med Internet Res. 2013;15: e85 10.2196/jmir.1933 23615206 10.2196/jmir.1933 PMC3636326

[23] Adams SA. Revisiting the online health information reliability debate in the wake of “web 2.0”: An inter-disciplinary literature and website review. International Journal of Medical Informatics. 2010;79: 391–400. 10.1016/j.ijmedinf.2010.01.006 20188623 10.1016/j.ijmedinf.2010.01.006

[24] Frost JH, Massagli MP. Social Uses of Personal Health Information Within PatientsLikeMe, an Online Patient Community: What Can Happen When Patients Have Access to One Another’s Data. Journal of Medical Internet Research. 2008;10: e15 10.2196/jmir.1053 18504244 10.2196/jmir.1053 PMC2553248

[25] Griffiths F, Dobermann T, Cave JA, Thorogood M, Johnson S, Salamatian K, et al The impact of online social networks on health and health systems: a scoping review and case studies. Policy & Internet. 2015;7: 473–496. 27134699 10.1002/poi3.97 PMC4841174

[26] Kata A. A postmodern Pandora’s box: Anti-vaccination misinformation on the Internet. Vaccine. 2010;28: 1709–1716. 10.1016/j.vaccine.2009.12.022 20045099 10.1016/j.vaccine.2009.12.022

[27] Kennedy A, LaVail K, Nowak G, Basket M, Landry S. Confidence About Vaccines In The United States: Understanding Parents’ Perceptions. Health Affairs. 2011;30: 1151–1159. 10.1377/hlthaff.2011.0396 21653969 10.1377/hlthaff.2011.0396

[28] Witteman HO, Zikmund-Fisher BJ. The defining characteristics of Web 2.0 and their potential influence in the online vaccination debate. Vaccine. 2012;30: 3734–3740. 10.1016/j.vaccine.2011.12.039 22178516 10.1016/j.vaccine.2011.12.039

[29] Betsch C, Renkewitz F, Haase N. Effect of Narrative Reports about Vaccine Adverse Events and Bias-Awareness Disclaimers on Vaccine Decisions: A Simulation of an Online Patient Social Network. Medical Decision Making. 2013;33: 14–25. 10.1177/0272989X12452342 22875721 10.1177/0272989X12452342

[30] Eysenbach G, Powell J, Englesakis M, Rizo C, Stern A. Health related virtual communities and electronic support groups: systematic review of the effects of online peer to peer interactions. Bmj. 2004;328: 1166 10.1136/bmj.328.7449.1166 15142921 10.1136/bmj.328.7449.1166 PMC411092

[31] Laranjo L, Arguel A, Neves AL, Gallagher AM, Kaplan R, Mortimer N, et al The influence of social networking sites on health behavior change: a systematic review and meta-analysis. Journal of the American Medical Informatics Association. 2014; 10.1136/amiajnl-2014-002841 25005606 10.1136/amiajnl-2014-002841 PMC4433372

[32] Maher CA, Lewis LK, Ferrar K, Marshall S, De Bourdeaudhuij I, Vandelanotte C. Are health behavior change interventions that use online social networks effective? A systematic review. J Med Internet Res. 2014;16: e40 10.2196/jmir.2952 24550083 10.2196/jmir.2952 PMC3936265

[33] Yang Q. Are Social Networking Sites Making Health Behavior Change Interventions More Effective? A Meta-Analytic Review. Journal of Health Communication. 2017;22: 223–233. 10.1080/10810730.2016.1271065 28248623 10.1080/10810730.2016.1271065

[34] Laukka E, Rantakokko P, Suhonen M. Consumer-led health-related online sources and their impact on consumers: An integrative review of the literature. Health Informatics Journal. 2017; 146045821770425. 10.1177/1460458217704254 28464727 10.1177/1460458217704254

[35] Ziebland S, Wyke S. Health and Illness in a Connected World: How Might Sharing Experiences on the Internet Affect People’s Health?: Does Sharing on the Internet Affect People’s Health? Milbank Quarterly. 2012;90: 219–249. 10.1111/j.1468-0009.2012.00662.x 22709387 10.1111/j.1468-0009.2012.00662.x PMC3460203

[36] Bowling A. Mode of questionnaire administration can have serious effects on data quality. Journal of Public Health. 2005;27: 281–291. 10.1093/pubmed/fdi031 15870099 10.1093/pubmed/fdi031

[37] StataCorp. Stata Statistical Software: Release 14 College Station, TX: STataCorp LP; 2015.

[38] Rock CL. Multivitamin-multimineral supplements: who uses them? Am J Clin Nutr. 2007;85: 277S–279S. 10.1093/ajcn/85.1.277S 17209209 10.1093/ajcn/85.1.277S

[39] Holman DM, Berkowitz Z, Guy GP, Hawkins NA, Saraiya M, Watson M. Patterns of sunscreen use on the face and other exposed skin among US adults. J Am Acad Dermatol. 2015;73: 83–92.e1. 10.1016/j.jaad.2015.02.1112 26002066 10.1016/j.jaad.2015.02.1112 PMC4475428

[40] Centers for Disease Control. FastStats: Oral and Dental Health [Internet]. [cited 1 Jan 2017]. Available: http://www.cdc.gov/nchs/fastats/dental.htm

[41] Centers for Disease Control. FastStats: Influenza [Internet]. [cited 1 Jan 2017]. Available: http://www.cdc.gov/nchs/fastats/flu.htm

[42] Hamm MP, Chisholm A, Shulhan J, Milne A, Scott SD, Given LM, et al Social media use among patients and caregivers: a scoping review. BMJ Open. 2013;3: e002819 10.1136/bmjopen-2013-002819 23667163 10.1136/bmjopen-2013-002819 PMC3651969

[43] Bejarano HD, Kaplan H, Rassenti S. Dynamic optimization and conformity in health behavior and life enjoyment over the life cycle. Frontiers in Behavioral Neuroscience. 2015;9 10.3389/fnbeh.2015.00137 26136666 10.3389/fnbeh.2015.00137 PMC4468389

[44] Eysenbach G, Powell J, Kuss O, Sa E-R. Empirical studies assessing the quality of health information for consumers on the world wide web: a systematic review. JAMA. 2002;287: 2691–2700. 12020305 10.1001/jama.287.20.2691

[45] Cole J, Watkins C, Kleine D. Health Advice from Internet Discussion Forums: How Bad Is Dangerous? Journal of Medical Internet Research. 2016;18: e4 10.2196/jmir.5051 26740148 10.2196/jmir.5051 PMC4720952

[46] Aral S, Walker D. Creating Social Contagion Through Viral Product Design: A Randomized Trial of Peer Influence in Networks. Management Science. 2011;57: 1623–1639. 10.1287/mnsc.1110.1421

[47] Nyhan B, Reifler J, Richey S, Freed GL. Effective Messages in Vaccine Promotion: A Randomized Trial. PEDIATRICS. 2014;133: e835–e842. 10.1542/peds.2013-2365 24590751 10.1542/peds.2013-2365

[48] Nyhan B, Reifler J. Does correcting myths about the flu vaccine work? An experimental evaluation of the effects of corrective information. Vaccine. 2015;33: 459–464. 10.1016/j.vaccine.2014.11.017 25499651 10.1016/j.vaccine.2014.11.017

[49] Centola D. An Experimental Study of Homophily in the Adoption of Health Behavior. Science. 2011;334: 1269–1272. 10.1126/science.1207055 22144624 10.1126/science.1207055

[50] Manski CF. Identification of Endogenous Social Effects: The Reflection Problem. The Review of Economic Studies. 1993;60: 531–542. 10.2307/2298123

[51] Çelen B, Kariv S, Schotter A. An Experimental Test of Advice and Social Learning. Management Science. 2010;56: 1687–1701. 10.1287/mnsc.1100.1228

[52] Çelen B, Kariv S. Distinguishing Informational Cascades from Herd Behavior in the Laboratory. American Economic Review. 2004;94: 484–498. 10.1257/0002828041464461

[53] Offerman T, Schotter A. Imitation and luck: An experimental study on social sampling. Games and Economic Behavior. 2009;65: 461–502. 10.1016/j.geb.2008.03.004

[54] McDonnell Norms Group. Antibiotic Overuse: The Influence of Social Norms. Journal of the American College of Surgeons. 2008;207: 265–275. 10.1016/j.jamcollsurg.2008.02.035 18656057 10.1016/j.jamcollsurg.2008.02.035

[55] Gentzkow M, Shapiro JM. What Drives Media Slant? Evidence From U.S. Daily Newspapers. Econometrica. 2010;78: 35–71. 10.3982/ECTA7195

[56] Gentzkow M, Shapiro JM. Ideological Segregation Online and Offline. The Quarterly Journal of Economics. 2011;126: 1799–1839. 10.1093/qje/qjr044

