# Supplementary material for: How does anonymous online peer communication affect prevention behavior? Evidence from a laboratory experiment
Source: PLoS One. 2018 Nov 21;13(11):e0207679. doi: 10.1371/journal.pone.0207679 (PMC6248974; doi:10.1371/journal.pone.0207679)
Supplement: S1 Table — Table indicates sample size within each of the experimental treatment groups. There was also a cross-cutting randomization where some subjects received a public health message about prevention. All subjects received the following prompt: “[]..you will be playing this game for 15 periods. In each period you will earn an income of US$ 10 if you stay healthy, and a income of zero if you fall sick. The probability of falling sick in each period is constant at p = [x] (a [x] in 10 chance) over the game period. In each period you can invest in a preventive health technology for $1 which lowers the probability of falling sick.” (DOCX) [file pone.0207679.s002.docx]

**S1 Table. Summary of experimental treatment arms**

|  | **Baseline Illness Probability** | | | **Total** |
| --- | --- | --- | --- | --- |
| *Technology effectiveness* | **p=0.3** | **p=0.5** | **p=0.7** |  |
| Prevention Technology Risk Reduction=0.12 | 193 | 75 | 89 | 357 |
| Prevention Technology Risk Reduction=0.20 | 159 | 77 | 86 | 322 |
|  |  |  |  |  |
| Total | 352 | 152 | 175 | 679 |

Table indicates sample size within each of the experimental treatment groups. There was also a cross-cutting randomization where some subjects received a public health message about prevention. All subjects received the following prompt: *“[]..you will be playing this game for 15 periods. In each period you will earn an income of US$ 10 if you stay healthy, and a income of zero if you fall sick. The probability of falling sick in each period is constant at p=[x] (a [x] in 10 chance) over the game period. In each period you can invest in a preventive health technology for $1 which lowers the probability of falling sick*.”
