# Supplementary material for: How does anonymous online peer communication affect prevention behavior? Evidence from a laboratory experiment
Source: PLoS One. 2018 Nov 21;13(11):e0207679. doi: 10.1371/journal.pone.0207679 (PMC6248974; doi:10.1371/journal.pone.0207679)
Supplement: S2 Table — All messages were coded into one of these four categories. Some messages were counted as both “informational” and either “encourages prevention” or “discourages prevention.” (DOCX) [file pone.0207679.s003.docx]

**S2 Table. Examples of Types of Messages Sent**

|  |  | Examples of Messages |
| --- | --- | --- |
| **Informational** | 1 | I invested and did not get sick |
|  | 2 | healthy again after investing. 4/6 healthy total - invested all times. it must just be me drinking lots of orange juice though - tech C doesn't seem to be that good. |
|  | 3 | yeah i did not invest and got sick twice but was also sick once when I invested |
|  |  |  |
| **Conversational** | 1 | brady has 3 kids now and is older. he def is at the end of his career |
|  | 2 | I really fancy putting cheese on my fries and throwing it in the microwave for a delicious 30seconds |
|  | 3 | not makin much this go 'round |
|  |  |  |
| **Encourages Prevention** | 1 | invest. Don't think about it just do it. In the long run you come out ahead. |
|  | 2 | It worked again. On the whole I think it's a good investment even if it sometimes doesn't work. |
|  | 3 | if you invest and get sick it's only -1 so if you at least win one the lest you make is $18. 1/4 |
|  |  |  |
| **Discourages Prevention** | 1 | this is friggin rediculous investing in this technology is a waste of money |
|  | 2 | i realize your gonna fall sick if your gonna fall sick..buying meds just gets you broke and sick |
|  | 3 | this is useless. we are all going to get sick and die. |

All messages were coded into one of these four categories. Some messages were counted as both “informational” and either “encourages prevention” or “discourages prevention.”
