# Supplementary figures and images for: How does anonymous online peer communication affect prevention behavior? Evidence from a laboratory experiment
Source: PLoS One. 2018 Nov 21;13(11):e0207679. doi: 10.1371/journal.pone.0207679 (PMC6248974; doi:10.1371/journal.pone.0207679)

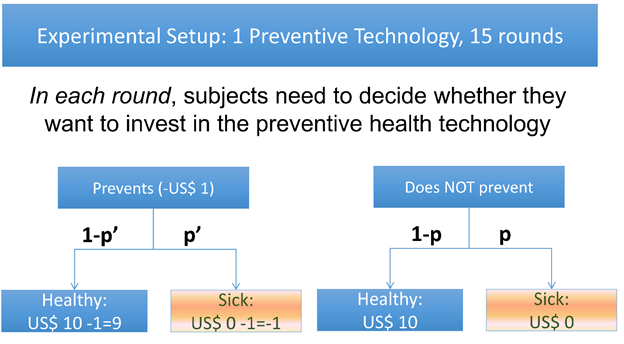

Supplement: S1 Fig — The probability p that a person fell ill when they did not prevent (p) was either 0.3, 0.5 or 0.7. The probability that a person fell when they prevented (p’) was lower than the probability of falling ill when they didn’t prevent (p’<p). (TIF) [file pone.0207679.s004.tif]

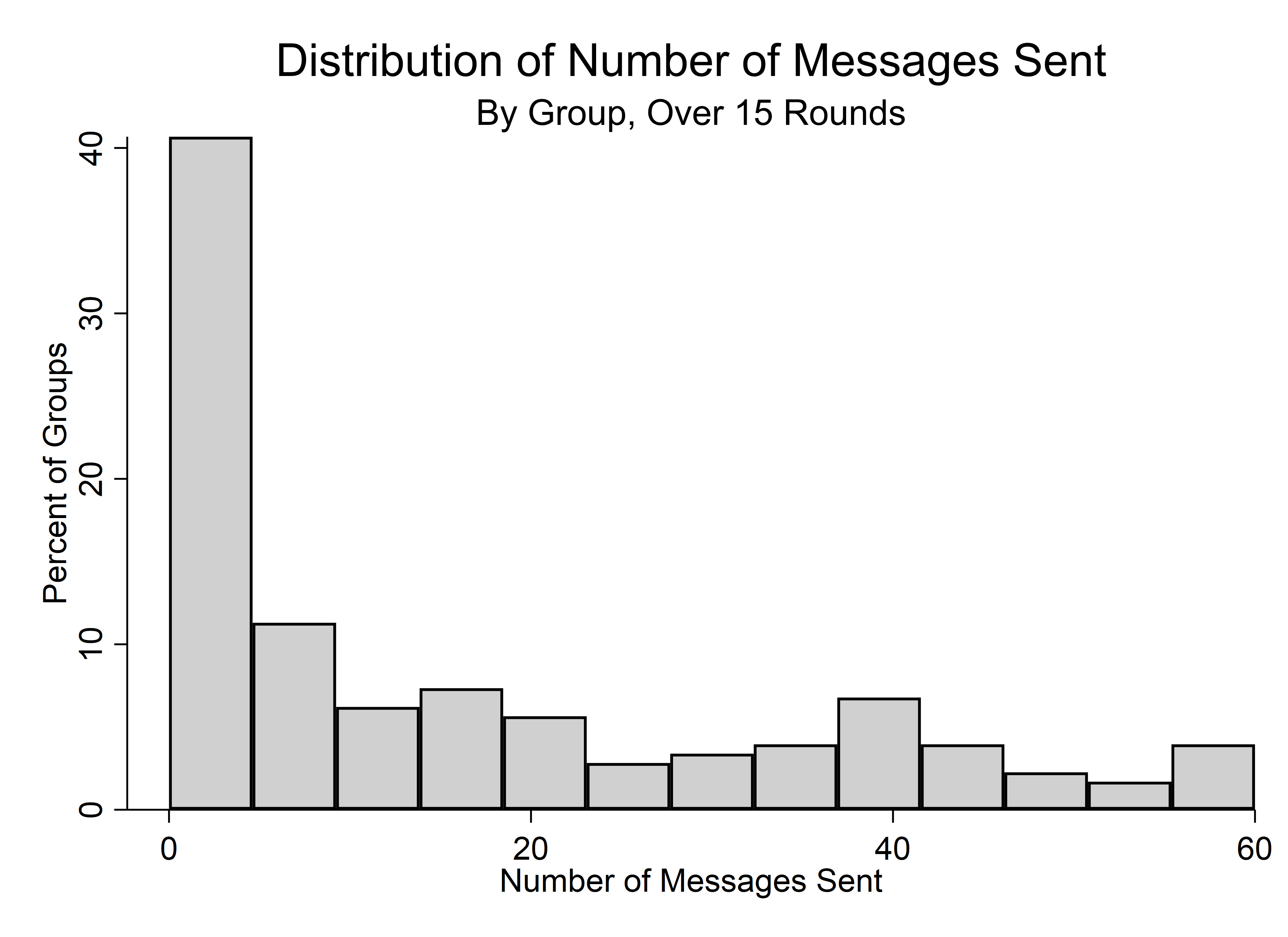

Supplement: S2 Fig — Distribution of total number of messages sent by all individuals in a group (N = 4) over 15 rounds. (TIF) [file pone.0207679.s005.tif]
